# The Role of Calprotectin in the Diagnosis and Treatment of Inflammatory Bowel Disease

**DOI:** 10.3390/ijms26051996

**Published:** 2025-02-25

**Authors:** Wenqian Wang, Wenfu Cao, Shenyun Zhang, Dapeng Chen, Lihong Liu

**Affiliations:** 1 Department of Physiology, College of Basic Medical Sciences, Dalian Medical University, Dalian 116044, China; 2 Comparative Medicine Department of Researching and Teaching, Dalian Medical University, Dalian 116044, Chinazhang6940007243@163.com (S.Z.)

**Keywords:** inflammatory bowel disease, fecal calprotectin, serum calprotectin, gastrointestinal disease, microbiota

## Abstract

The management of inflammatory bowel disease (IBD), which is characterized by immunodeficiency, has attracted increasing attention, highlighting the necessity for more precise and streamlined diagnostic approaches in clinics. Calprotectin, an immune cell-derived protein with inherent anti-inflammatory and antimicrobial properties, plays a pivotal role in immune regulation and intestinal homeostasis. Its expression levels are intricately linked to IBD activity, enabling differentiation between inflammatory and non-inflammatory states while predicting recurrence risks. As a non-invasive biomarker, fecal calprotectin (FC) and serum calprotectin (SC) analysis offers high reproducibility and clinical utility, facilitating both IBD diagnosis and real-time disease monitoring. Beyond its diagnostic specificity in distinguishing IBD from other gastrointestinal disorders, calprotectin also emerges as a promising therapeutic target, due to its dual role in modulating inflammatory pathways and interacting with the gut microbiota. With collaborative advancements in standardized detection protocols and innovative research methodologies, it is anticipated that calprotectin-based strategies will be integrated into mainstream clinical practice for IBD.

## 1. Introduction

IBD, encompassing ulcerative colitis (UC) and Crohn’s disease (CD), represents a group of chronic immune-mediated disorders affecting the gastrointestinal tract, impacting an estimated 6 to 8 million individuals globally [1]. Patients with IBD commonly experience gastrointestinal symptoms like diarrhea, abdominal pain, and bloody stools, alongside systemic manifestations, such as fever, appetite loss, anemia, weight loss, and fatigue [2]. Despite advances in therapeutic strategies (e.g., biologics, immunosuppressants, immunomodulators, and biologics), current regimens predominantly target symptomatic relief rather than disease modification, reflecting persistent gaps in understanding IBD pathogenesis [3,4]. A critical challenge concerns diagnostics, namely the heterogeneity of IBD phenotypes, the overlap with other enteropathies, and the limitations of invasive gold-standard techniques like colonoscopy, particularly its inability to visualize the small intestine adequately, which underscore the urgent need for the identification of non-invasive biomarkers [5,6].

Recent attention has focused on FC, a calcium-binding protein released by neutrophils and macrophages during intestinal inflammation. As a biomarker of innate immune activation, FC exhibits high diagnostic accuracy in differentiating IBD from functional gastrointestinal disorders (pooled sensitivity 91%, specificity 90% in meta-analyses) [7]. Its non-invasive nature and correlation with endoscopic disease activity have positioned FC as a tool for monitoring treatment response and predicting relapse [8]. Investigating the biological functions of calprotectin in IBD may yield novel insights for its diagnosis and treatment, underscoring the relevance of research on calprotectin’s role in this condition. Nevertheless, there still lacks a more systematic and clearer summary of the role of calprotectin in IBD. This study aims to sum up the role of calprotectin through a rigorous examination of calprotectin’s mechanistic roles in IBD pathophysiology. Our findings clarify the biomarker function of calprotectin in both IBD and other gastrointestinal disorders, and further provide mechanistic insights to guide the development of calprotectin-targeted therapies. Moreover, a detailed understanding of the biological function of calprotectin in IBD can provide new ideas for the diagnosis and treatment of IBD in the future. Therefore, this study on the role of calprotectin in IBD has certain practical significance.

## 2. Calprotectin: Physicochemical Properties and Pathophysiological Data

Calprotectin, a 36-kDa protein and a member of the S100 family, comprises calcium-binding proteins characterized by two EF hands (helix–loop–helix motifs), a central hinge, and N- and C-terminal structural domains. The nomenclature “S100” stems from their solubility in 100% ammonium sulfate at a neutral pH [9]. Structurally, calprotectin encompasses multiple amino acid residues folded into a compact three-dimensional configuration, enabling it to sustain biological activity and execute vital functions in diverse environments. Each subunit of calprotectin (S100A8 and S100A9) possesses two distinct calcium-binding sites, thereby bolstering the stability and functionality of calprotectin [10].

Calprotectin, primarily derived from neutrophils and macrophages, serves as a potent neutrophil chemotactic factor. During inflammation, neutrophils are mobilized to migrate to the inflammatory site, triggering degranulation and the substantial release of calprotectin [11]. While calprotectin is typically absent in macrophages within normal tissues, it may be discharged by macrophages during intestinal mucosal inflammation, albeit to a lesser extent than by neutrophils [12]. A study of IBD confirmed that calprotectin levels were significantly elevated in the stools of patients with inflammatory bowel disease, and the degree of elevation was positively correlated with the severity of the disease [13]. Notably, calprotectin exhibits antibacterial and anti-inflammatory properties, directly influencing inflammation regulation and mitigation at the site of inflammation. Meanwhile, calprotectin can impact the proliferation, differentiation, and function of immune cells, thereby modulating the body’s immune response. Additionally, in certain instances, calprotectin can function as an antigen-presenting molecule, participating in the initiation and regulation of immune responses [14]. A study on calprotectin highlighted that, acting as an immunomodulatory protein, calprotectin can influence the function of the intestinal immune system and, consequently, indirectly impact the homeostasis of the intestinal flora [15].

In individuals with IBD, a persistent inflammatory cascade occurs within the gastrointestinal tract, resulting in an escalation of neutrophil presence. Upon activation, these neutrophils undergo degranulation, releasing various inflammatory mediators, including calprotectin [16]. Consequently, calprotectin levels experience a significant surge in IBD patients [17]. Throughout the active phase of IBD, intensified intestinal inflammation leads to a further influx of neutrophils, thereby sustaining elevated calprotectin levels. Conversely, during the remission phase of IBD, as intestinal inflammation subsides, both neutrophil counts and calprotectin levels decrease correspondingly [18]. In essence, the levels of calprotectin in individuals with IBD exhibit a positive correlation with disease severity. Schoepfer AM et al. found that in adults, calprotectin levels below 50 μg/g indicated a negative status for IBD, while levels above 600 μg/g indicated that the disease was in a severely active phase [19]. In contrast, in children with a calprotectin level below 600 μg/g and lacking symptoms indicative of IBD, the likelihood of IBD diagnosis is low [20].

**Figure 1:**
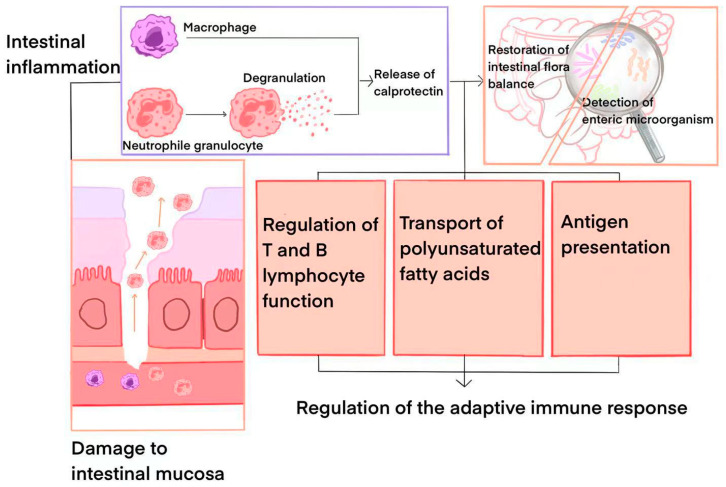
Calprotectin’s involvement in the pathophysiological process of IBD.

When the intestinal tract becomes irritated or infected, calprotectin stimulates the secretion of anti-inflammatory mediators [21], thereby reducing intestinal inflammation. This anti-inflammatory effect plays a crucial role in preserving the integrity of the intestinal barrier function and preventing barrier damage as a result of inflammation. In individuals that do not have IBD, the intestinal mucosal barrier remains intact, limiting the entry of neutrophils and other inflammatory cells into the intestinal lumen. In contrast, patients with IBD experience damage to the intestinal mucosal barrier due to erosions and ulcers, leading to a substantial increase in the influx of inflammatory cells into the intestinal lumen and, consequently, escalating levels of calprotectin in fecal samples. Within the intestines of individuals with IBD, the persistent inflammatory response triggers an increase in the number and activation of immune cells, such as neutrophils and macrophages, resulting in markedly elevated calprotectin levels. Calprotectin plays a role in inducing neutrophil chemotaxis, promoting their migration and activation, thereby enhancing the body’s ability to combat infections. Moreover, macrophages have the capacity to release calprotectin and interact with other immune cells through this protein. In Carnazzo V’s study, it was found that macrophages may regulate the inflammatory response and intestinal barrier function through calprotectin-mediated signaling pathways [22]. Interestingly, they found that calprotectin also exerts an influence on the function of T cells and B cells, thereby regulating the body’s adaptive immune response. Additionally, calprotectin can serve as an antigen-presenting molecule in certain scenarios, participating in the initiation and regulation of adaptive immune responses [22]. The immunomodulatory role of calprotectin in IBD extends to maintaining immune system homeostasis and facilitating the transport of polyunsaturated fatty acids to inflammatory sites to stimulate local immune responses [23]. Moreover, calprotectin significantly impacts the gut microbiota, as studies indicate that calprotectin deficiency in mice alters their gut microbiota composition, rendering them more susceptible to various diseases [24]. In the context of IBD, calprotectin may help restore the gut flora balance by regulating the function of intestinal immune cells and detecting microbial changes within the gut more effectively [25]. Figure 1 shows how calprotectin is involved in the pathophysiological process of IBD.

## 3. Calprotectin in the Diagnosis of IBD

### 3.1. Diagnostic Value

Enteroscopy stands as the conventional method for diagnosing IBD, yet its invasiveness, the requirement for bowel preparation, and potential discomfort during the examination, including symptoms like nausea, vomiting, and even hematemesis, mean that it is not an optimal method [26]. In contrast, FC testing offers the advantages of being non-invasive, convenient, and reproducible [27].

In addition, FC demonstrates exceptional accuracy in diagnosing IBD and its subtypes, with FC currently being the sole biomarker endorsed and recommended by the European Crohn’s and Colitis Organisation [28]. Notably, a significant correlation exists between FC and ultrasound in assessing the severity of intestinal inflammation in pediatric patients and the simple endoscopic score [29]. In terms of the accuracy of FC in detecting IBD, a study involving 139 patients who underwent a diagnostic ileocolonoscopy revealed significantly higher fecal levels of lactoferrin (Lf), Cal, PMN-e, and serum CRP in individuals with UC or CD with active inflammation compared to those with inactive inflammation and patients with irritable bowel syndrome (IBS) (all *p* < 0.05). The overall diagnostic accuracy of FC in IBD patients reached 80%, performing notably well in regard to UC (83.3%). In another clinical investigation comparing FC and Lf in UC patients, FC slightly outperformed Lf [30]. In studies on the specificity and sensitivity of FC, we can see a further increase in the sensitivity and specificity of the combined activity index, which results in a diagnostic accuracy of up to 95.3% in patients with UC. Meanwhile, it was observed that an FC threshold value of ≤50 μg/g exhibited superior sensitivity compared to a value >50 μg/g (87% vs. 79%), with sensitivity decreasing and specificity increasing as the critical value increases [31]. This dynamic reflection of changes in intestinal inflammatory activity in UC patients allows for real-time monitoring. In an international multicenter study, combining FC with C-reactive proteins facilitated the assessment of upadacitinib’s efficacy and safety as an induction therapy for pediatric UC or IBD-U, with patients achieving normal C-reactive protein levels and FC values < 150 mcg/g reaching remission rates of 75% and 50%, respectively. The available data indicated the combined remission of CFR and FC levels [32]. Only IL-6 levels surpassed inactive UC in differentiating between active and inactive UC and exhibited a positive correlation with FC (*p* = 0.021) [33]. In a separate prospective single-center cohort study, endoscopic (MES = 1) and histologic (GS < 2) remission were compared to SC and CRP, with only FC being able to fully detect these outcome markers, while SC proved valuable in distinguishing between patients in remission and those with UC, due to its convenience [34].

In basic experiments, FC levels were significantly elevated in mice with colitis caused by changes in their circadian rhythm [35]. Similarly, SC showed high specificity and sensitivity in distinguishing between idiopathic IBD dogs and healthy dogs [36]. For patients with CD, FC and serum CRP can guide disease management in both asymptomatic and symptomatic cases. In instances of discordance between symptom assessment and biomarker values, endoscopic evaluation may be required to confirm the disease activity status [37]. Even so, FC screening for suspected IBD cases can still reduce unnecessary endoscopy. Given that the occurrence of multiple inflammatory bowel diseases is age related and is correlated with changes in the microbiota, models combining FC and serological scores show greater accuracy [38]. Systemic inflammatory markers like CRP, TNF-α, and IL-6 [39] can also aid IBD diagnosis and monitoring, although they generally lack the sensitivity and specificity of FC. FC better reflects intestinal inflammatory activity in IBD and correlates effectively with endoscopic scores [40]. FC demonstrates a sensitivity of 91.1% and specificity of 86.7% in differentiating between IBD and IBS, with an overall test accuracy of 88.9%. Another study corroborated FC’s superior accuracy in predicting endoscopic and histologic activity among 10 distinct inflammatory biomarkers [41]. In a study involving children and adolescents with IBD, calprotectin levels notably decreased with a decreasing Pediatric Ulcerative Colitis Activity Index score, but not CRT [42]. Additionally, in a clinical investigation involving pediatric CD patients, the fecal myeloperoxidase (MPO) activity and fecal myeloperoxidase (fMPO) protein concentrations were found to be quasi-predictive of CD diagnosis and active disease, correlating with FC (r = 0.78, *p* < 0.0001, and r = 0.81, *p* < 0.0001), yet FC outperformed MPO activity and fMPO protein concentration in predicting CD and the extent of disease-related inflammation [43]. Fecal characterization based on the microbial load, water content, calprotectin concentration, and bowel type may aid in selecting biologic therapies for IBD treatment [44], predicting treatment efficacy in IBD patients. Apart from FC, several other biomarkers, such as lactoferrin and myeloperoxidase [45], can be utilized in IBD diagnosis. Each biomarker possesses distinct advantages and disadvantages, yet FC stands out in terms of IBD diagnosis due to its high sensitivity, specificity, non-invasiveness, and convenience. Combining calprotectin with markers like MPO, the human neutrophil lipid transport protein, the eosinophil cationic protein, and the eosinophil-derived neurotoxin, enhances FC’s diagnostic power [46]. Notably, treatment with common drugs like nonsteroidal anti-inflammatory drugs or proton pump inhibitors can impact the specificity of FC [47]. A similar issue arose in another clinical trial, where the correlation between calprotectin and disease vanished in UC patients receiving a daily 3 g dose of anthocyanin AC-rich lingonberry extract [48]. Hence, in the era of personalized medicine, identifying and monitoring biomarkers for specific therapies remain crucial.

### 3.2. Role in Disease Surveillance and Prognostic Assessment

FC, as a non-invasive and convenient biomarker, enables the indirect quantitative assessment of intestinal inflammation, effectively monitoring disease activity in IBD [46]. In patients with IBD, FC levels are closely linked to the severity of intestinal inflammation. The characterization of calcineurin can delineate the disease’s heterogeneity. Regular monitoring of fecal calcineurin levels allows physicians to stay updated on changes in IBD patients’ conditions, providing a crucial basis for adjusting treatment regimens and prescribing appropriate medications [49]. For example, in clinical settings, combining FC concentrations with the microbial load, water content, and fecal bowel type characterization can aid in selecting biologic therapies for IBD treatment [44]. While endoscopy is the gold standard for the diagnosis of IBD and the assessment of mucosal healing, studies have shown that FC is very useful in assessing disease activity, predicting recurrence, and guiding treatment decisions. FC can also be used as a reference point for assessing mucosal healing, effectively reducing the physical burden caused by the need for frequent endoscopies [50]. In the realm of targeted therapies, the development of non-invasive markers for assessing mucosal healing in UC patients holds paramount importance. Following a study involving 61 UC patients, of whom 79% exhibited endoscopic healing, researchers discovered that the combination of a normal bowel ultrasound, the absence of rectal bleeding, and an FC value of <172 µg/g identified mucosal healing in all the patients. Utilizing a bowel ultrasound in conjunction with FC proves effective in evaluating mucosal healing in UC patients, offering a non-invasive means of assessment for most individuals [51]. Calprotectin also significantly contributes to the development of anti-inflammatory and pro-mucosal healing drugs. In in vivo whole-animal studies, zinc pyrithione potentially exerts anti-inflammatory and pro-mucosal healing effects via TRP channels in the IEC, thereby demonstrating anti-colitis properties and ameliorating DSS-induced colitis symptoms [52].

**Figure 2:**
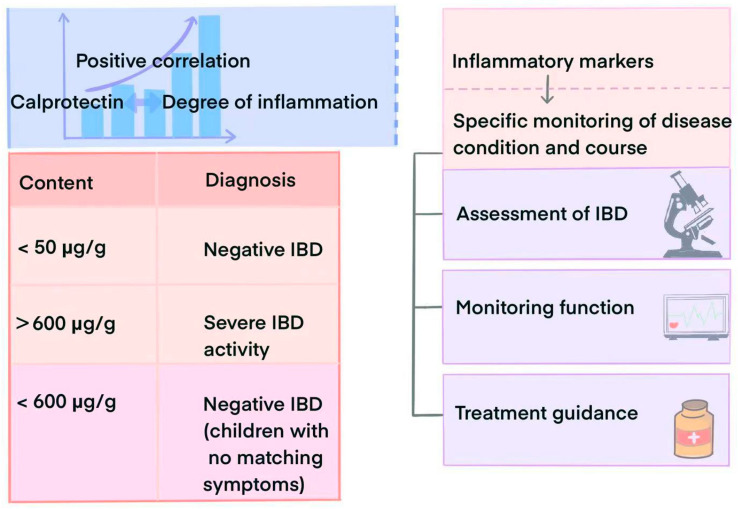
The application of calprotectin in the diagnosis of IBD.

Researchers investigating the likelihood of clinical relapse in Crohn’s disease patients observed that in their multivariate model encompassing the Crohn’s Disease Endoscopic Index of Severity, FC, high-sensitivity C-reactive protein (hsCRP), and histology, only FC exhibited a predictive role for clinical recurrence in patients with mucosal healing (*p* = 0.029). This precision in regard to residual inflammation assessment provided by FC offers more accurate and targeted interventions compared to random biopsies. Despite investigators proposing a high FC threshold of 153 μg/g, it still demonstrated a high level of sensitivity (96%) in predicting histological remission [53]. Furthermore, FC levels have shown a strong association with the risk of IBD complications, like intestinal obstruction and perforation [54]. Notably, SC combined with other blood biomarkers (e.g., CRP or albumin) can effectively diagnose and differentiate between IBD and CD, potentially predicting disease onset and outcomes [55]. By monitoring fecal and serum calprotectin levels, physicians can anticipate the risk of recurrence and complications in IBD patients, enabling more proactive treatment measures to mitigate patient risks. Additionally, Lan et al. introduced a novel calprotectin assay, a split luciferase calprotectin assay, facilitating the convenient detection of physiologically relevant calprotectin levels in various sample types, including serum, plasma, and whole blood [56]. Calprotectin, thus, assumes a crucial role in both diagnosing and predicting IBD, offering novel avenues for clinical testing. Figure 2 illustrates the application of calprotectin in the diagnosis of IBD.

## 4. The Potential Value of Calprotectin in the Treatment of IBD

### 4.1. Potential as a Therapeutic Target

Calprotectin, due to its pivotal role in IBD, has emerged as a promising therapeutic target. Recently, Chen et al. introduced a delivery system, a calcium tungstate functional microgel (CTM), designed to specifically target calprotectin. Leveraging the robust interaction between Ca^2+^ and calprotectin, CTM can transport probiotics to sites exhibiting high inflammatory gene expression. By suppressing the growth of *Enterobacteriaceae* bacteria, CTM effectively mitigates intestinal inflammation, restores the intestinal barrier function, and, ultimately, achieves therapeutic outcomes in colitis patients [57]. Modulating the levels or functions of calprotectin may offer avenues to reduce intestinal inflammation, enhance the intestinal barrier function, and rebalance the immune system. Research has explored calcineurin’s role in regulating the metabolism and the proliferation of intestinal zinc in a mouse jejunal organoid epithelium [58]. While the targeted treatment of IBD through calprotectin manipulation is still under scrutiny, Chen et al.’s study presents a novel approach in this realm of investigation.

### 4.2. Evaluation of Treatment Effects

Following effective treatment, FC levels decrease in tandem with the subsiding intestinal inflammation [59]. This alteration serves as a pivotal indicator for evaluating treatment efficacy. A sustained decrease in FC post-treatment signifies effective therapy and the alleviation of intestinal inflammation [60]. Conversely, persistent elevation or stability of FC levels post-treatment may necessitate treatment regimen adjustments or the exploration of alternative options [61]. In terms of clinical treatment, in cases where clinical symptoms like abdominal pain, diarrhea, and rectal bleeding diminish in IBD patients alongside a decline in FC levels, the treatment can be deemed effective. Conversely, if symptoms persist or worsen while FC levels remain high or unchanged, reassessment and modification of the treatment plan may be warranted [62]. Moreover, FC serves as a valuable tool in predicting the risk of relapse in IBD patients [63]. Regular monitoring of FC levels enables physicians to identify shifts in the patient’s condition and implement appropriate therapeutic interventions to mitigate the risk of relapse and enhance the patient’s quality of life.

## 5. Relationship Between Calprotectin and Microbiota in IBD

Inflammatory bowel disease, a multifaceted intestinal disorder, encompasses bacteria, viruses, fungi, and protozoa, with the gut microbiota playing a pivotal role in its pathogenesis [64]. Dysregulation of the gut microbiota is a key factor in the progression of IBD, and many studies, to date, have highlighted the complex interplay between gut microbiota, immune modulation, and anti-inflammatory responses [65]. Calcineurin has been utilized as a non-invasive marker for IBD, with recent research revealing correlations between various intestinal microorganisms and FC. Cibeira et al. identified associations between *Escherichia coli* and *Methanobrevibacter smithii* with FC in patients with Crohn’s disease with active inflammation, while *Ruminococcus* sp. correlated with FC in ulcerative colitis patients, aiding in the differentiation between CD and UC [66]. Conversely, another study indicated a negative correlation between FC in patients with IBD who were in remission and the virulence-related enzyme lipase of Candida albicans [67]. In a recent double-blind randomized controlled trial, Jessica Breton et al. observed negative correlations between FC and changes in the relative abundance of bifidobacteria (q = 0.08) and anaerobic bacteria (q = 0.017) [68]. Baston et al. highlighted the presence of the C4 genotype of the Mycobacterium genus in all UC patients compared to 18.1% of healthy controls, with FC showing a correlation with the C4 genotype, while the CRP values did not differ significantly [69]. FC exhibited positive correlations with *Lachnospiraceae*, *Blautia*, and *Bacteroides*, and negative correlations with *Enterobacteriaceae*, *Veillonella*, *Akkermansia*, and *Escherichia* levels in individual patients [70]. Early microbiome alterations may precede clinical symptoms and changes in FC levels, potentially indicating increased disease activity in patients with IBD [71]. The intricate relationship between FC and the gut microbiome in IBD patients underscores the need for comprehensive studies to elucidate the differences in bacterial imbalances across various gut diseases, influenced by age, diet, lifestyle habits, and mental health. Notably, combining FC with microbial markers and other indicators (e.g., CRP, hemoglobin Hb, IgA) proves effective in distinguishing between IBD subtypes and active versus remitting patients [72].

## 6. Application of Calprotectin in Regard to Other Gastrointestinal Diseases

Fecal biomarkers are independent predictors of IBD-related outcomes [73]. Calcineurin plays a crucial role not only in diagnosing, monitoring, and assessing treatment efficacy in IBD, but also in various other gastrointestinal disorders. In anorexia nervosa, calcineurin can act as an indicator of the intestinal inflammatory process, with elevated fecal calcineurin levels correlating with the duration of the illness [74]. Combining calcineurin testing with fecal whole-blood testing enables the identification of symptomatic patients at high risk of colorectal cancer, aiding physicians in prioritizing those requiring an urgent colonoscopy [75]. Studies on colorectal tumors have shown that patients with adenomas and colorectal cancer exhibit higher levels of FC and Lf compared to healthy individuals [29]. As a non-invasive marker, FC demonstrates good validity and accuracy in predicting stenosis post-necrotizing enterocolitis [76]. Elevated FC levels in cirrhosis patients can assist in the early detection of chronic liver disease, potentially reducing sepsis incidence and mortality rates [77]. In patients with nonalcoholic fatty liver disease, FC levels serve as an effective marker for distinguishing between UC and IBS patients, aiding in therapeutic decision making [78]. Plasma calprotectin serves as a crucial indicator for intensive care transfer in suspected sepsis cases [79], while serum calprotectin proves valuable in assessing the inflammatory phase in patients with immune-mediated gastrointestinal disorders [80]. Urinary calprotectin can function as a diagnostic tool for severe bacteriuria detection, enhancing the accuracy of urinary tract infection screening through purulent urine test paper examinations [81]. In a clinical prospective study, FC accurately differentiated between Crohn’s disease patients with active luminal disease and those that did not have the disease, with significantly higher FC levels observed in Crohn’s disease patients compared to those with cryptoglandular fistulas [82]. Moreover, calprotectin can serve as a marker of intestinal inflammation in cystic fibrosis patients [83] and aid in distinguishing between organic and functional enteropathy [84,85]. Furthermore, calprotectin has emerged as a specific marker for various gastrointestinal diseases in animal studies, showing effectiveness in monitoring cats with chronic enteropathy [86]. In addition, FC can serve as a topographic biomarker of CD activity due to its superior sensitivity in identifying increased mucosal inflammation from proximal to distal [87]. Similarly, FC is a promising non-invasive biomarker for assessing disease severity and long-term outcomes in patients with Celiac Disease and Non-Celiac Enteropathies [88]. Table 1 describes some of the disorders associated with elevated calprotectin levels.

## 7. Conclusions and Outlook

FC serves as a widely utilized adjunctive diagnostic indicator for IBD, demonstrating high sensitivity and specificity in distinguishing organic IBD from functional disorders like IBS [89]. When diagnosing IBD, FC exhibits superior sensitivity and accuracy compared to other fecal markers (e.g., CRP, fMPO, Lf, IL-6, SC, etc.). However, the impact of drug therapy on FC sensitivity or its loss as a marker necessitates consideration, underscoring the importance of identifying therapy-specific surveillance markers in modern personalized medicine. Conversely, calprotectin has exhibited notable accuracy in disease prognosis and monitoring. FC, as the only biomarker capable of predicting recurrence in clinical trials, displays enhanced accuracy and comprehensiveness compared to histological scores observed endoscopically. Several clinical studies have highlighted the unique responsiveness of FC in monitoring patients’ disease status promptly, despite potential sensitivity reductions due to drug therapy. Recent research has increasingly focused on combining FC with other biomarkers (e.g., fecal myeloperoxidase, C-reactive protein, etc.) [90] to enhance the precision and predictability of IBD diagnosis. Furthermore, investigations have commenced to explore the distinctions and specificity of FC among different subtypes of IBD, such as Crohn’s disease and ulcerative colitis [35]. Moreover, recent findings indicate that targeting calprotectin can offer specificity in colitis treatment. Using the multifunctional properties of calcium tungstate microgels to target areas with high expression of calprotectin, reduce inflammation and restore intestinal barrier function, it provides a new avenue for IBD drug development [57]. As research attention increasingly shifts towards the gut microbiota of IBD patients, a strong correlation has been observed between FC and various microbes. These microbial markers can characterize patients’ conditions, indicating the complex relationship between FC and gut microbes, and the need for further investigations to elucidate their causal links with the disease. FC can function as a specific marker in regard to other gastrointestinal disorders, such as uremia and other organic intestinal conditions, aiding in distinguishing IBD from diseases like IBS.

Future research demands thorough investigations into the specific mechanisms of calprotectin in the pathogenesis and progression of IBD, encompassing its involvement in intestinal inflammation and interactions with other inflammatory mediators. This exploration is essential for unraveling the onset of IBD, identifying novel treatment targets, and fostering innovative therapeutic approaches. Furthermore, there is a pressing need to explore more sensitive, specific, convenient, rapid, and cost-effective calprotectin assays that can be efficiently integrated into clinical practice. Emphasizing the importance of clinical studies, it is crucial to establish a scientifically sound and effective FC threshold to accurately differentiate between various IBD subtypes and other intestinal disorders, which would offer valuable insights for clinical management. Simultaneously, integrating FC with markers like SC and fMPO to enhance the precision and efficiency of IBD diagnosis will advance the evolution of personalized medicine. The precise role of calprotectin in immune function remains ambiguous, necessitating further exploration into how calprotectin influences intestinal microbes to restore the intestinal barrier, potentially presenting a novel avenue for leveraging calprotectin to modulate intestinal microbes for IBD treatment. A deeper comprehension of calprotectin’s mechanisms could pave the way for developing novel therapeutic drugs or methods targeting calprotectin or its associated pathways in the future. These innovations, combined with small molecules like gels and herbal monomers, are poised to significantly impact the effective management of IBD and other gastrointestinal disorders.

Methods:

1. Search Strategy:

A systematic search was conducted in PubMed (January 2018 to December 2024) using the combined keywords: (“calprotectin” OR “S100A8/A9”) AND (“IBD” OR “inflammatory bowel disease” OR “Crohn’s disease” OR “ulcerative colitis” OR “gastrointestinal disorders”) AND (“therapy” OR “treatment” OR “diagnosis” OR “management”). Medical Subject Headings (MeSH) were activated to optimize retrieval.

2. Screening Criteria:

Inclusion: original research articles, randomized controlled trials (RCTs), and cohort studies focusing on calprotectin’s mechanistic roles or clinical applications in IBD.

Exclusion:

(i) Review articles, meta-analyses, and commentaries with prior synthesized evidence to avoid redundancy (n = 298 excluded);
(ii) Studies lacking primary data on calprotectin–IBD interactions (n = 378 excluded);
(iii) Publications that were not peer reviewed (e.g., conference abstracts, preprints).

3. Data Extraction:

Two authors independently screened the titles/abstracts using Covidence^®®^ software (https://www.covidence.org/, accessed on 11 September 2024), resolving discrepancies through consensus. The full texts of 458 potentially relevant articles were assessed, with 90 meeting the eligibility criteria.

4. Limitations:

While our focus on using articles contained within PubMed ensured depth was achieved in regard to the biomedical literature, we acknowledge that potential omissions may have occurred in relation to other databases like Embase or Web of Science. To mitigate this, reference lists of the included studies were hand searched to identify additional relevant works.

## Figures and Tables

**Table 1:** Other diseases with elevated calprotectin levels.

Name	Level (μg/g, Median)	Contribution	Ref.	Calprotectin	Species
Anorexia Nervosa	273.5 (124–423)	Dina Moubayed et al.	[74]	FC	Human
Colorectal Cancer	117.3 (26.5–390.8)	Ángel Lanas et al.	[75]	FC	Human
Colorectal Tumors	279 (28–1536)	Tsukasa Yamakawa et al.	[30]	FC	Human
Stenosis Post-necrotizing Enterocolitis	1237.55 (741.25–1378.80)	Guanglin Chen et al.	[76]	FC	Human
Cirrhosis	543.5 (207.09–879.91)	Dinesh Jothimani et al.	[77]	FC	Human
Sepsis	2.2 mg/L	Åsa Parke et al.	[79]	SC	Human
Severe Bacteriuria	1758.5 (242.4–6260.4)	Sabina Waldecker-Gall et al.	[81]	Urinary calprotectin	Human
Active Luminal Disease	1746.0 (741.8–1800.0)	Zhou Z et al.	[82]	FC	Human
Chronic Enteropathy	61 (3–1066)	Romy M Heilmann et al.	[86]	FC	Cat
Celiac Disease	250.0 mg/kg (111.9–416.0)	Annalisa Schiepatti et al.	[88]	FC	Human

## Abbreviations

The following abbreviations are used in this manuscript:

IBD	Inflammatory bowel disease
FC	Fecal calprotectin
SC	Serum calprotectin
UC	Ulcerative colitis
CD	Crohn’s disease
Lf	Lactoferrin
IBS	Irritable bowel syndrome
MPO	Myeloperoxidase
fMPO	Fecal myeloperoxidase
CRP	C-reactive protein
CTM	Calcium tungstate functional microgel
